# Acceptance and perceived usefulness of digital health services in the management of chronic urticaria: a survey of patients and physicians

**DOI:** 10.1186/s12913-025-13043-7

**Published:** 2025-07-02

**Authors:** Michael Hindelang, Alexander Zink, Johannes Knitza, Robert Darkow, Martin Welcker, Tilo Biedermann, Susann May, Felix Muehlensiepen

**Affiliations:** 1 TUM School of Medicine and Health, Department of Dermatology and Allergy, Technical University of Munich, Munich, Germany; 2 Pettenkofer School of Public Health, Munich, Germany; 3 Institute for Medical Information Processing, Biometry and Epidemiology (IBE), Faculty of Medicine, Ludwig-Maximilian University, LMU Munich, Munich, Germany; 4 Institute for Digital Medicine, University Hospital of Giessen and Marburg, Philipps University Marburg, Marburg, Germany; 5 FH| JOANNEUM Gesellschaft mbH, Graz, Austria; 6 MVZ for Rheumatology Dr. Martin Welcker GmbH, Planegg, Germany; 7 Center for Health Services Research, Faculty of Health Sciences, Brandenburg Medical School Theodor Fontane, Rüdersdorf, Germany; 8 Deutsches Herzzentrum der Charité - Department of Cardiology, Angiology and Intensive Care Medicine, Charitéplatz 1, Berlin, 10117 Germany; 9 Charité - Universitätsmedizin Berlin, corporate member of Freie Universität Berlin and Humboldt-Universität zu Berlin, Charitéplatz 1, Berlin, 10117 Germany; 10 Université Grenoble Alpes, AGEIS, Grenoble, France

**Keywords:** Chronic urticaria, Dermatology, Digital health, User perspectives, Technology acceptance, Barriers to adoption

## Abstract

**Background:**

Chronic urticaria (CU) is a complex and unpredictable skin condition that significantly affects patients’ quality of life. As the healthcare landscape increasingly integrates digital health technologies, understanding their perceived usefulness in CU management from both patient and physician perspectives is crucial.

**Objective:**

This study investigates the acceptance, perceived usefulness, and potential barriers to using digital health services, such as medical apps and video consultations, among patients with CU and their healthcare providers.

**Methods:**

A quantitative survey was conducted across multiple specialized centers, specialist clinics, and general practices, involving both patients and physicians. The study utilized standardized questionnaires to assess digital health literacy, technology readiness, and attitudes toward adopting digital health services in CU management. Descriptive and inferential statistics, including Fisher’s exact test, were employed to analyze the data.

**Results:**

A substantial proportion of the 121 surveyed patients and 101 physicians perceived digital health technologies as beneficial in managing CU, with 59.5% of patients and 75.3% of physicians agreeing on their advantages. However, 21.5% of patients and 14.9% of physicians remained neutral, while 8.3% of patients and 4.0% of physicians found these technologies unhelpful. Key barriers to adoption were identified, including concerns over data privacy, limitations in technical infrastructure, and a lack of awareness of available digital health solutions.

**Conclusion:**

While many patients and physicians recognize the potential of digital health technology to improve urticaria management, some remain uncertain or skeptical. Addressing concerns and improving digital understanding is critical to the future implementation and integration of these technologies into care. Due to the cross-sectional design of the study and the self-reported data, further research may be needed to confirm these results.

## Summary box

### What is already known on this topic

- Digital health services (DHS), such as medical apps and video consultations, have the potential to enhance disease management in chronic conditions like chronic urticaria (CU).
- Acceptance and adoption of DHS vary significantly, with healthcare providers generally showing higher enthusiasm compared to patients.
- Barriers such as data privacy concerns, technical infrastructure, and low digital health literacy impede the broader adoption of DHS.

### What this study adds

- This cross-sectional study highlights that while most patients and physicians perceive DHS as beneficial for CU management, physicians exhibit significantly higher readiness and enthusiasm for technology adoption.
- The study identifies specific barriers to adoption, including patients’ low confidence in decision-making based on digital health information and physicians’ concerns about cost and insufficient evidence of efficacy.
- Patients show a preference for synchronous communication (e.g., video consultations) but still value traditional in-person appointments, especially for first consultations or emergencies.

### How this study might affect research, practice or policy

- The findings underscore the need for targeted strategies to improve digital health literacy among patients and enhance their confidence in using DHS effectively.
- Policymakers and healthcare providers should focus on robust data protection measures and evidence-based promotion of DHS to address both patients’ and physicians’ concerns.
- Integrating DHS into CU care requires balancing technological efficiency with the need for personal interaction to ensure patient-centered care.

## Introduction

Chronic urticaria is a skin disease characterized by the appearance of itchy wheals or hives. Angioedema also occurs in up to 40% of cases [1–4]. We speak of chronic urticaria when the disease lasts longer than 6 weeks. One of the biggest challenges with chronic urticaria is that flare-ups can occur very quickly and unpredictably. These flare-ups can be a significant burden for sufferers. This disease significantly impairs the quality of life and productivity of patients with chronic urticaria and leads to more absences from work [2, 5].

Urticaria can lead to sleep problems, difficulties in everyday activities, and a strong increase in anxiety and stress [6–8]. Angioedema is swelling of the skin that may affect the skin surface or deeper skin layers and mucous membranes. There may be rapid and unpredictable swelling. This can cause a feeling of suffocation [8–10].

This type of swelling occurs in about 40% of people with urticaria [11]. Up to 67% of people with CU have both angioedema and wheals at the same time [12]. For this reason, the guideline-based treatment of urticaria includes a specific escalation algorithm to achieve complete symptom control [4]. A large global observational study (AWARE) has shown that people with antihistamine-refractory CU often have uncontrolled symptoms, are prone to angioedema or even comorbid chronic induced urticaria, and their quality of life is severely affected, relying on many medical resources [13]. Despite clear guideline recommendations, AWARE revealed that only a minority of patients receive appropriate escalation therapy, with many remaining on ineffective treatments for prolonged periods. Another important study, ASSURE-CSU, highlighted the substantial economic and humanistic burden of CSU, demonstrating that undertreatment not only leads to increased healthcare utilization but also significantly impacts work productivity and daily functioning. Other studies have reported that there is a pattern of undertreatment and that guidelines are not properly followed [3, 7, 14, 15].

Treatment of CU usually requires an interdisciplinary team. Dermatologists and general practitioners are crucial, but sometimes, depending on the situation, specialists from other fields such as allergy are also needed [3]. The diagnostic process can be quite involved. Therefore, it is often better for patients to contact specialized centers or clinics that combine all the necessary expertise. Patients themselves can contribute to a timely diagnosis by documenting the course of the disease, therapies, skin changes, nutrition, etc. in a urticaria diary (including photo documentation). Such documentation, such as a urticaria diary (and possibly photos), can be very helpful in diagnosing and treating the disease [16–20].

Despite the existence of specialized UCARE centers and various participation opportunities in the diagnostic and therapeutic process, achieving a timely diagnosis and optimal symptom control remains challenging due to the unpredictable nature of CU, which significantly burdens the affected individuals [7]. This situation presents numerous opportunities for the implementation of digital health services in CU management [21, 22]. The newest innovative approaches using digital tools, including teledermatology, mobile health apps, and remote monitoring tools like wearables or chatbots, could improve access to care and support self-management [23–26]. Studies show that teledermatology reduces travel burdens and enhances timely medical advice, while CU tracking apps improve physician-patient communication [18–20, 27]. Before these concepts can be implemented in real world practice, it is essential to assess the willingness of patients and physicians. The opportunities and obstacles associated with these implementations must be thoroughly analyzed.

## Objectives

This study aims to assess the level of acceptance of digital health services among CU patients and healthcare providers. It also seeks to identify which digital health services are currently being utilized in CU management and to determine the specific areas of care where these services are applied. Furthermore, the study explores the opportunities and barriers experienced by both healthcare providers and patients in using digital health services to manage CU.

## Methods

### Study design

This study was designed as a cross-sectional survey to evaluate the acceptance, perceived usefulness, and barriers to using digital health services in managing CU.

### Setting

The study was conducted across multiple outpatient clinics and practices in Germany specializing in CU management. These included dermatology practices, specialized urticaria centers (UCARE centers), and general medical practices. The recruitment and data collection phases took place over a specified period from February 2023 to November 2023.

### Participants

Patients eligible for inclusion were individuals aged 18 years or older who had already been diagnosed with CU by a specialist, either a dermatologist or an allergist/immunologist, prior to their participation in the study. Healthcare providers included in the study were dermatologists, general practitioners, and assistant physicians who were actively involved in the management of chronic urticaria (CU). While we did not specifically assess formal training in CU management, all participating physicians had direct experience in managing CU patients. Additionally, we collected demographic data on physicians, including age, to examine potential variations in responses based on age. Our subgroup analysis did not reveal significant differences in responses due to the age of the physicians. We performed a stratified analysis based on the number of patients treated per physician, and no significant differences were found regarding digital health service use. Exclusion criteria included patients without a confirmed urticaria diagnosis, individuals without proficiency in German, minors, and healthcare providers not directly involved in urticaria care. Participants were recruited from specialized urticaria centers, dermatology practices, and general medical practices. Participants completed the questionnaires individually in a controlled, private setting, either in their healthcare provider’s office or a designated area within the clinic, to minimize any external influence.

### Variables

The primary variables included participants’ attitudes toward digital health services, frequency of use, and perceived barriers to adoption. We also assessed the impact of COVID-19 on digital health service utilization. CU was defined according to EAACI guidelines as the appearance of wheals, angioedema, or both, lasting more than six weeks [4]. Demographic data such as age, gender, place of residence, and income were collected. Place of residence was categorized into five groups: large city (> 100,000), medium city (20,000–100,000), small town (5,000–20,000), rural area (< 5,000), and million city (> 1,000,000). Income was grouped into less than €2,500, between €2,500 and €5,000, and more than €5,000. We also measured technology readiness and digital health literacy using the G-eHEALS scale, a validated instrument for assessing individuals’ ability to seek, appraise, and apply health information from digital sources. The G-eHEALS captures key dimensions of eHealth literacy, including confidence in navigating online health resources and evaluating their credibility. Additionally, we examined the perceived impact of digital services on the doctor-patient relationship, care quality, and changes in usage due to the COVID-19 pandemic [28, 29].

### Data sources and measurement

The self-developed questionnaire was first tested in a pilot study to assess clarity, reliability, and validity. A diverse sample of nine participants, comprising five patients and four physicians, provided feedback to ensure representation of both patient and healthcare provider perspectives. The gender distribution of participants was 3 male and 6 female. Participants were asked to complete the questionnaire and comment on item wording, comprehension, and overall structure. Based on their feedback, minor adjustments were made to enhance the clarity of certain questions and improve response options where needed. Data were collected from both patients and healthcare providers using two standardized questionnaires. The patient questionnaire assessed digital health literacy, employing the G-eHEALS scale, and technology readiness, using an adaptation from the Technology Acceptance Model 2. The physician questionnaire similarly examined the professional use and perceptions of digital health services. Both questionnaires used Likert scales for response measurement and dichotomous options for specific behaviors. To ensure comparability between patients and physicians, the questionnaires included parallel items addressing technology readiness and digital service utilization.

### Bias

Efforts to mitigate bias included the use of standardized and validated questionnaires. Sensitivity analyses were performed to evaluate the impact of missing data and potential variations in responses across different demographic groups, further ensuring the reliability of the findings.

### Quantitative variables

In this study, quantitative variables were derived from standardized questionnaires that captured various aspects of digital health service utilization and attitudes toward digital technologies. Key variables include technology readiness, digital health literacy, frequency of digital health service usage, and perceived impact on the doctor-patient relationship and quality of care. Responses were measured using Likert scales and dichotomous choices to assess agreement levels and behaviors. Socio-demographic data, such as age, gender, and professional background, were also collected to inform subgroup analyses.

### Statistical methods

Descriptive statistics were used to summarize the demographic and baseline characteristics of the participants. Categorical variables were presented as frequencies and percentages. For continuous variables, mean values and standard deviations were calculated. Group comparisons were performed using chi-square or Fisher’s exact tests for categorical variables and independent t-tests or Mann-Whitney U tests for continuous variables. Statistical significance was defined as a p-value of less than 0.05.

Data analysis was performed using R Statistical Software (Version 2023.06.2; R Core Team, 2023). The analysis involved key packages such as dplyr [30] for data manipulation, Likert [31], and ggplot2 [32] for data visualization, and appropriate statistical testing functions available within base R.

### Subgroup analysis

Subgroup analyses were conducted to explore potential differences in responses based on demographic factors, such as age, gender, and professional role (e.g., dermatologists versus general practitioners). The relationship between technology readiness and digital health service utilization was also examined across subgroups to identify trends and variations. These analyses aimed to provide a more granular understanding of how specific groups perceive and interact with digital health services.

**Fig. 1:**
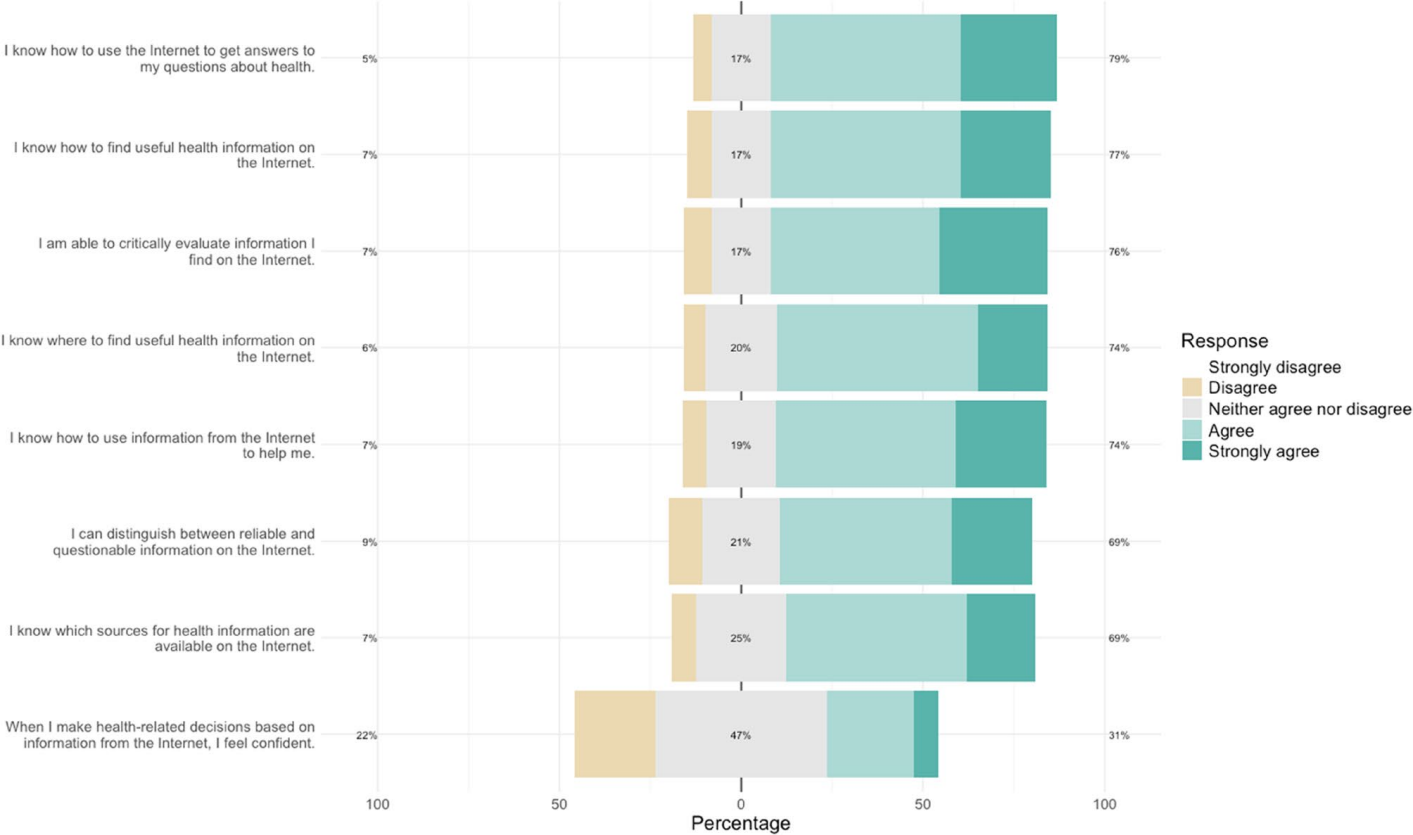
Devices, media, and health services used or offered in urticaria care (Patients *n* = 121; Physicians *n* = 101): This figure shows the percentage of patients (blue) and physicians (orange) using or offering digital tools for urticaria care. Smartphones, the Internet, and email were most used, while video consultations and digital health applications (DiGA) were less common. Physicians more frequently engaged in self-administered blood sampling and online pharmacies

Another subgroup analysis (Fig. 1) revealed that patients predominantly used digital apps rather than video consultations. This is consistent with data from another study [21], which also showed higher usage of apps compared to video consultations. This pattern may reflect a genuine preference for in-person visits, but it could also be due to the current lack of a dedicated digital platform for urticaria care. In another study, we implemented and evaluated a digital platform for urticaria and demonstrated that remote monitoring via patient-reported outcomes is feasible [26]. Furthermore, our recent systematic review on chatbots for medical history-taking [23] underscores the transformative potential of digital tools to streamline clinical workflows and enhance patient engagement, reinforcing the need for dedicated digital platforms.

In this study, missing data were handled by including only observations where at least 80% of the data were complete. Observations with more than 20% of missing data were excluded from the analysis to maintain the robustness and reliability of the findings.

Sensitivity analyses tested the robustness of findings by exploring response variations based on key demographics like age and gender. Two approaches addressed uncertain responses: first, by grouping ‘Agree/Strongly Agree (including Neutral)’ to capture broader acceptance, and second, by excluding neutral responses to focus on clear agreement or disagreement.

## Results

### Participant characteristics

A total of 121 patients with CU and 101 physicians participated in the study (Tables 1 and 2). The median age of patients was 40.5 years (SD = 15.1), with 64.5% (78/121) identifying as female, 18.2% (22/121) as male, and 16.5% (20/121) not providing a gender. Among physicians, the median age was 36 years (SD = 12.8), with 56.4% (57/101) identifying as female, 34.7% (35/101) as male, 1.0% (1/101) as diverse, and 7.9% (8/101) not providing a gender. A significant difference in gender distribution was observed between patients and physicians (*p* = 0.0179).

**Table 1:** Demographics

Category	Patients *n* = 121 Frequency (%)	Physicians *n* = 101 Frequency (%)
Age Category		
Under 30	18 (14.9)	9 (8.9)
30–39	28 (23.1)	37 (36.6)
40–49	12 (9.9)	15 (14.9)
50–59	25 (20.7)	10 (9.9)
60 and above	11 (9.1)	13 (12.9)
Missing	27 (22.3)	17 (16.8)
Median Age (SD)	40.5 (15.1)	36 (12.8)
Gender		
Diverse	1 (0.8)	1 (1.0)
Male	22 (18.2)	35 (34.7)
Female	78 (64.5)	57 (56.4)
Missing	20 (16.5)	8 (7.9)
Educational Status		
I am still a student	5 (4.1)	-
I am still in vocational training	5 (4.1)	-
Without vocational training completion	2 (1.7)	-
Completion of vocational training of at least one year	37 (30.6)	-
University degree	49 (40.5)	-
Missing	23 (19.0)	-
Place of Residence		
Large City (> 100,000)	17 (14.0)	-
Small Town (5,000–20,000)	21 (17.4)	-
Rural Area (< 5,000)	21 (17.4)	-
Million City (> 1,000,000)	22 (18.2)	-
Medium City (20,000–100,000)	19 (15.7)	-
Missing	21 (17.4)	-
Income		
Up to 850 Euro	7 (5.8)	-
851–1,500 Euro	9 (7.4)	-
1,501–2,000 Euro	11 (9.1)	-
2,001–2,750 Euro	25 (20.7)	-
2,751–3,500 Euro	19 (15.7)	-
3,501–5,000 Euro	12 (9.9)	-
5,001–10,000 Euro	5 (4.1)	-
More than 10,000 Euro	0 (0.0)	-
Missing	33 (27.3)	-
eHEALS		
Low (< = 20)	9 (7.4)	-
Moderate (21–32)	78 (64.5)	-
High (> 32)	34 (28.1)	-
eHEALS questionnaire (Agreement)		
I know which sources of health information are available on the Internet. (Missing 4)	83 (68.6)	-
I know where to find useful health information on the Internet. (Missing 3)	90 (74.4)	-
I know how to find useful health information on the Internet. (Missing 4)	93 (76.9)	-
I know how to use the Internet to find answers to my health-related questions. (Missing 3)	95 (78.5)	-
I know how to use information from the Internet in a way that helps me. (Missing 0)	90 (74.4)	-
I am able to critically evaluate information I find on the Internet. (Missing 0)	92 (76.0)	-
I can distinguish between reliable and questionable information on the Internet. (Missing 1)	84 (69.4)	-
When making health-related decisions based on information from the Internet, I feel confident. (Missing 6)	37 (30.6)	-

**Table 2:** Physicians’ characteristics

Professional activity	Physicians *n* = 101 Frequency (%)
Assistant Physician (Other Hospital)	5 (5.0)
Assistant Physician (Private Practice)	7 (6.9)
Assistant Physician (University Hospital)	23 (22.8)
General Practitioner (Private Practice)	10 (9.9)
Dermatologist (Other Hospital)	4 (4.0)
Dermatologist (Private Practice)	24 (23.8)
Dermatologist (University Hospital)	18 (17.8)
Other	2 (2.0)
Missing	8 (7.9)
How many patients do you treat on average per quarter?	
up to 500	22 (21.8%)
500 to 1,500	35 (34.6%)
more than 1,500	27 (26.7%)
Missing	17 (16.8%)
How many patients with urticaria do you treat on average per quarter?	
0–10	41 (40.6%)
11–50	21 (20.8%)
51–200	18 (17.8%)
> 200	2 (2.0%)
Missing	19 (18.8%)
Do you think your patients (urticaria treatment) find digital health services useful?	
I don’t know	24 (24.8)
Yes	59 (63.4)
No	10 (10.8)
N/A	8 (7.9)

### Digital health literacy and technology readiness

Most of the patients demonstrated moderate or higher levels of digital health literacy (Fig. 1), according to the eHealth Literacy Scale (eHEALS). Specifically, 7.4% of patients (9/121) fell into the “Low” category, 64.5% (78/121) were in the “Moderate” category, and 28.1% (34/121) were classified as having “High” eHealth literacy (Table 1). This indicates that 71.9% (87/121) of patients exhibited at least a moderate ability to find and use health information online. Over 75% (93/121) of patients knew how to use the Internet to find health-related information. However, only 30.6% (37/121) felt confident making health decisions based on online information.

Physicians showed greater enthusiasm for technological innovations than patients (Table 3). Specifically, 80.2% (81/101) of physicians were very curious about new technologies compared to 60.3% (73/121) of patients (*p* = 0.002), and 55.4% (56/101) of physicians desired to use technological products more frequently versus 27.3% (33/121) of patients (*p* < 0.001).

**Table 3:** Technology readiness among patients and physicians; presents the percentage of patients and physicians who agreed with each statement regarding their technology readiness. Percentages are based on the total number of respondents per group (Patients: *n* = 121, physicians: *n* = 101)

Question	Frequency (in %) Patients	Frequency (in %) Physicians	*p* value
Whether I am successful in using modern technology largely depends on me.	76 (62.8)	57 (56.4)	0.408
It is up to me whether I succeed in using technological innovations – it has little to do with luck.	79 (65.3)	63 (62.4)	0.757
I am very curious about technological innovations.	73 (60.3)	81 (80.2)	0.002
What happens when I engage with technological innovations is ultimately under my control.	64 (52.9)	61 (60.4)	0.324
I quickly develop a liking for technological innovations.	62 (51.2)	66 (65.3)	0.048
When I have difficulties with technology, it ultimately depends on me to solve them.	46 (38.0)	35 (34.7)	0.705
I am always interested in using the latest technological devices.	47 (38.8)	61 (60.4)	0.002
If I had the opportunity, I would use technological products even more frequently than I do currently.	33 (27.3)	56 (55.4)	0.000
Dealing with technological innovations is often overwhelming for me.	16 (13.2)	12 (11.9)	0.923
I often fear failing when dealing with modern technology.	14 (11.6)	11 (10.9)	1.000
I find it difficult to handle new technology – I usually just can’t do it.	14 (11.6)	7 (6.9)	0.344
I am afraid of breaking technological innovations rather than using them correctly.	8 (6.6)	14 (13.9)	0.115

### Attitudes toward digital health services

Regarding the usefulness of digital health services in managing urticaria, 59.5% (72/121) of patients and 75.3% (76/101) of physicians agreed or strongly agreed (Table 4). While 59.5% of patients and 75.3% of physicians agreed on the usefulness of these services, no significant demographic differences were observed in patient attitudes across age, gender, education, or place of residence (Table 5). However, patients from smaller towns and rural areas tended to show higher agreement rates compared to those from larger cities. While physicians showed a higher tendency to view these services favorably, the difference was not statistically significant (*p* = 0.091). Among patients, 21.5% (26/121) were neutral, and 8.3% (10/121) disagreed.

**Table 4:** Perceived Usefulness of Digital Health Services in Chronic Urticaria Management: Patient and Physician Responses

“Do you consider the use of digital health services (e.g., medical apps, video consultations) useful for managing urticaria?”			*p*-value
Response	Patients Frequency (%)	Physicians Frequency (%)	0.09124
Applies	72 (59.5)	76 (75.3)	
Neutral	26 (21.5)	15 (14.9)	
Does not apply	10 (8.3)	4 (4.0)	
Missing	13 (10.7)	6 (5.9)	

**Table 5:** Frequency and percentage of patients agreeing with the use of digital health services for managing urticaria by demographic group

“Do you consider the use of digital health services (e.g., medical apps, video consultations) useful for managing urticaria?”	Frequency (% of Patients in the category (agree/strongly agree))
Age Group	*p* = 0.617
Under 30	18 (50.0)
30–39	28 (67.9)
40–49	12 (75.0)
50–59	25 (64.0)
60 and above	11 (72.7)
Gender	*p* = 0.147
Female	78 (61.5)
Male	22 (81.8)
Educational Status	*p* = 0.554
Still in school	5 (40.0)
Still in vocational training	5 (80.0)
No vocational qualification	2 (50.0)
Vocational training of at least one year	37 (73.0)
University degree	49 (63.3)
Place of Residence	*p* = 0.187
Rural area (community under 5,000 inhabitants)	21 (76.2)
Small town (5,000–20,000 inhabitants)	21 (81.0)
Medium-sized town (20,000–100,000 inhabitants)	19 (68.4)
Large city (over 100,000 inhabitants)	17 (47.1)
Metropolitan city (over 1,000,000 inhabitants)	22 (59.1)

The COVID-19 pandemic positively influenced attitudes toward digital health services. Among patients, 29.8% (36/121) reported a more favorable view due to the pandemic, compared to 46.5% (47/101) of physicians (*p* = 0.049). Approximately one-third of both groups reported increased use of digital health services since COVID-19: 31.4% (38/121) of patients and 33.7% (34/101) of physicians.

### Usage of digital health services

Current usage patterns (Fig. 1) revealed that 51.2% of patients (62/121) used smartphones for urticaria care, and 56.2% (68/121) utilized the Internet. Physicians offered services involving smartphones and the Internet at rates of 43.6% (44/101) and 51.5% (52/101), respectively. Physicians expressed a higher intention to adopt digital health applications (“DiGA” in Germany) in the future compared to patients, with 24.8% (25/101) of physicians and 10.7% (13/121) of patients expressing interest (*p* = 0.0071). Video consultations were currently used by about 15% of both groups, with physicians having a higher rate of offering them before COVID-19 (18.8% [19/101] vs. 5.0% [6/121] of patients, *p* = 0.0013).

### Perceived impact of digitalization on care and preferences for video consultations

Regarding the doctor-patient relationship, 31.4% of patients (38/121) and 40.6% of physicians (41/101) perceived digitalization as having a positive or very positive impact (Table 6). Concerning the quality of care, 40.5% of patients (49/121) and 56.5% of physicians (57/101) believed digitalization had a positive effect. For future use of video consultations, 36.4% of patients (44/121) were willing to use them for follow-up appointments, and 18.2% (22/121) for first appointments. Physicians were more inclined to offer video consultations for follow-ups (51.5%, 52/101) but less so for emergencies compared to patients.

**Table 6:** Comparison of patients’ and physicians’ attitudes toward digital health services

	Patients Frequency (%) *n* = 121 (100)	Physicians Frequency (%) *n* = 101 (100)	*p*-value
Has your attitude towards digital health services changed due to COVID-19?			
Yes, it has become more positive	36 (29.75)	47 (46.5)	0.04891
No, it has not changed	65 (53.72)	42 (41.6)	
Yes, it has become more negative	6 (4.96)	6 (5.9)	
Missing	14 (11.57)	6 (5.9)	
Do you use more digital health services since COVID-19?” (Patients)/“Do you offer more digital health services since COVID-19? (Physicians)			
Yes	38 (31.4)	34 (33.7)	0.6072
No	70 (57.9)	60 (59.4)	
Missing	13 (10.7)	7 (6.9)	
How does digitalization in urticaria care affect the doctor-patient relationship?			
Very positive	14 (11.6)	8 (7.9)	0.1308
Rather positive	24 (19.8)	33 (32.7)	0.1337
Both positive and negative	25 (20.7)	32 (31.7)	0.261
Rather negative	12 (9.9)	9 (8.9)	0.5377
Very negative	2 (1.6)	2 (2.0)	1
Not at all	24 (19.8)	9 (8.9)	0.0005
Missing	20 (16.5)	8 (7.9)	0.002
How does digitalization in urticaria care affect the quality of care?			
Very positive	22 (18.2)	22 (21.8)	1
Rather positive	27 (22.3)	35 (34.7)	0.2085
Both positive and negative	25 (20.7)	19 (18.8)	0.2864
Rather negative	7 (5.8)	9 (8.9)	0.7244
Very negative	3 (2.5)	2 (2.0)	1
Not at all	16 (13.2)	6 (5.9)	0.006
Missing	21 (17.4)	8 (7.9)	0.0014
For which purpose/area would you use video consultations in the future?			
First appointment	22 (18.2)	12 (11.9)	0.0283
Follow-up appointment	44 (36.4)	52 (51.5)	0.3123
Not at all	19 (15.7)	21 (20.8)	0.8233
Emergency appointment	16 (13.2)	6 (5.9)	0.006
Other	5 (4.1)	4 (4.0)	1
Missing	15 (12.4)	6 (5.9)	0.0126
Would you be willing to forego an in-person appointment if your condition is stable and you could indicate that you are doing well using digital health services? If so, how?			
No, I prefer an in-person appointment, even if I am well and there is nothing to discuss	83 (68.6)	-	
Yes, digitally, preferably via phone call or video consultation (synchronous)	25 (20.7)	-	
Yes, digitally, preferably via digital questionnaire or email (asynchronous)	13 (10.7)	-	

### Advantages and barriers of digital health services

**Fig. 2:**
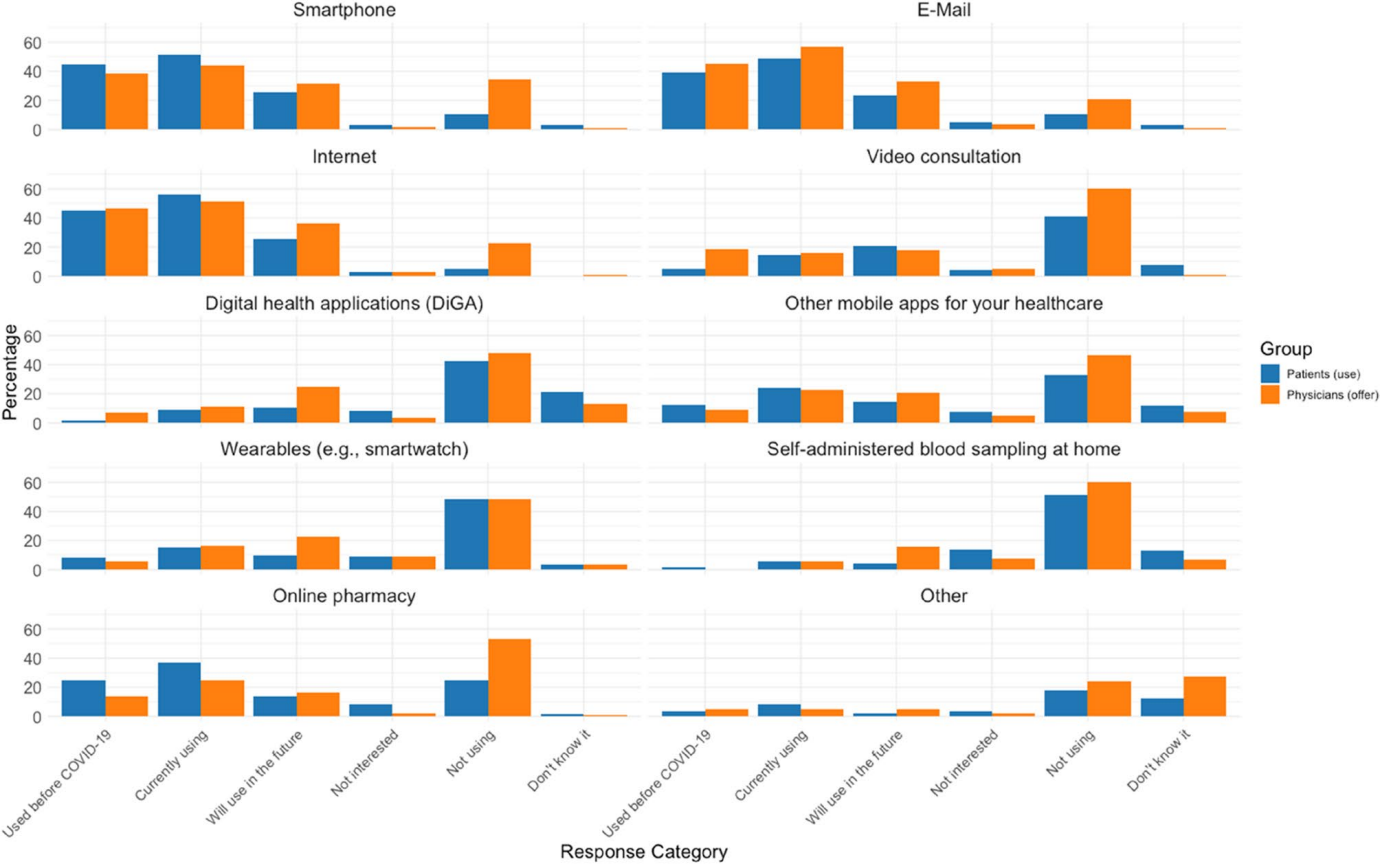
Advantages and barriers of digital health services

Both groups recognized advantages (Fig. 2) such as location-independent use and increased flexibility. Physicians more frequently cited benefits like detailed documentation of the disease course (40.6% [41/101] vs. 14.0% [17/121] of patients, *p* < 0.001) and better preparation for consultations (27.7% [28/101] vs. 14.9% [18/121], *p* = 0.021). Barriers identified included lack of knowledge among users, technical limitations, and data protection concerns. Physicians were more concerned about high costs (14.9% [15/101] vs. 4.1% [5/121] of patients, *p* = 0.011) and insufficient evidence of benefits (26.7% [27/101] vs. 9.1% [11/121], *p* = 0.001).

## Discussion

### Key results

This study assessed the acceptance, utilization, and perceived barriers of digital health services among patients with CU and their healthcare providers in Germany. The findings revealed that a significant majority of physicians (75.3%) and a substantial proportion of patients (59.5%) consider digital health services useful for managing CU. Physicians demonstrated higher enthusiasm and readiness to adopt technological innovations compared to patients, with 80.2% expressing curiosity about new technologies versus 60.3% of patients. Although physician caseloads may influence responses, our stratified analysis showed no significant impact on digital health service acceptance. Despite the positive attitudes, notable barriers were identified, including concerns about data privacy, technical infrastructure limitations, and a lack of awareness of available digital services. The COVID-19 pandemic positively influenced attitudes toward digital health services, particularly among physicians, with 46.5% reporting a more favorable view due to the pandemic compared to 29.8% of patients. Despite expectations, educational background did not significantly impact the use of digital health services (Appendix 1).

### Interpretation and comparison with literature

Our sample had a higher proportion of female participants, a trend seen in other studies on urticaria prevalence [3, 33, 34]. Nevertheless, our sensitivity analysis revealed no significant gender differences in the acceptance of digital health services.

The results indicate a growing recognition of the potential benefits of digital health services in CU management among both patients and physicians. The higher acceptance among physicians aligns with findings by Ruggiero et al., who reported increased enthusiasm for teledermatology among dermatologists, especially after the onset of the COVID-19 pandemic [35]. The pandemic has accelerated the adoption of digital health services. 46.5% of physicians in our study report a more positive attitude towards digital solutions due to COVID-19. This is consistent with the observations of Kruse, Monaghesh and Hajizadeh, who have seen a faster adoption of telemedicine across specialties [25, 36, 37]. 

Patients’ positive attitudes, though less pronounced than physicians’, are consistent with Cherrez-Ojeda et al., who found that patients with CU are increasingly open to digital tools for disease management [21]. Furthermore, a recent publication on the hybrid care potential of teledermatology in skin diseases demonstrated that linking digital and inperson care can enhance patient satisfaction and support disease management [25]. The critical evaluation of health information often requires specialist knowledge that is difficult for lay people to access. Our results show that only 30.6% of patients feel confident to make health-related decisions based on online information. This underlines the importance of trusted medical sources such as professional societies and specialized clinics to provide patients with reliable information. While in our study 22% stated that they felt unsure about making decisions based on online health information, in another study in the German general population [38] it is 32% and in a survey of people with rheumatic diseases 42.5% [39]. Our findings echoes Diviani et al., who highlighted that low health literacy impedes the ability to critically evaluate online health information [40]. Enhancing digital health literacy is crucial for empowering patients and facilitating the effective use of digital tools.

The identified barriers, such as data privacy concerns and technical infrastructure limitations, are consistent with those reported by Kruse et al. [41] in their systematic review of telemedicine adoption barriers. Our findings suggest higher acceptance of digital health services in small towns and rural areas, likely due to limited specialist access. Studies highlight teledermatology’s benefits in such regions, improving expert access for CU patients [42, 43]. Another study [26] further confirms strong patient interest, reinforcing its role in bridging healthcare gaps.

Physicians in our study were more likely to perceive high costs (14.9% vs. 4.1% of patients, *p* = 0.011) and insufficient evidence of benefits (26.7% vs. 9.1% of patients, *p* = 0.001) as significant obstacles. This aligns with concerns about the financial and evidential aspects of digital health implementation discussed by Ross et al. [44], emphasizing the need for robust clinical evidence and cost-effectiveness analyses to support digital interventions.

The preference for in-person visits among patients, even when their condition is stable (68.6% preferred to continue in-person visits), reflects a desire for personal interaction and trust in traditional consultations. Additionally, first appointments in CU often require in‑office challenge tests and hands‑on examination - essential for confirming diagnosis and guiding treatment - that cannot be performed remotely, thereby limiting digital services primarily to follow‑up care [45]. This is consistent with Moulaei et al. [46], who noted that despite the convenience of telemedicine, patients value the quality of care and personal connection inherent in face-to-face consultations.

However, mobile health (mHealth) tools are emerging as valuable solutions. The CRUSE^®^ study demonstrated their feasibility [18, 19], with Sousa-Pinto et al. [20]. confirming their reliability. Cherrez-Ojeda et al. [47]. found strong patient interest, particularly among younger individuals, while Sørensen et al. [48] showed that smartphone photographs can support remote assessment [20]. 

### Implications for practice

The higher enthusiasm among physicians suggests that they could play a pivotal role in promoting the adoption of digital health services. Their professional exposure to technological advancements and the necessity to adapt clinical practices during the pandemic position them as key facilitators in integrating digital tools into CU management. Engaging patients through education and addressing their concerns could bridge the gap between perceived usefulness and actual usage. Tailored interventions to improve digital health literacy, as recommended by Norman and Skinner [49], could enhance patients’ confidence in using online health information.

Addressing data privacy concerns is essential. Implementing robust data protection measures and clearly communicating these to patients can alleviate fears related to privacy and security [41]. Improving technical infrastructure, such as ensuring reliable internet access and providing updated equipment, is necessary to support the effective delivery of digital health services [50].

Providing evidence of the efficacy and cost-effectiveness of digital health interventions is crucial to encourage adoption among healthcare providers [51]. Physicians’ concerns about costs and insufficient evidence highlight the need for comprehensive studies demonstrating the clinical benefits and economic viability of digital health services [52, 53]. The ideal digital health intervention for improving the management of CU could involve a hybrid care model that combines mobile health apps, teledermatology, and remote monitoring through wearables or patient-reported outcomes. This integrated approach would allow for real-time symptom tracking, personalized care plans, and timely adjustments to treatment.

### Limitations and generalisability

This study has several limitations. First, its cross-sectional design captures attitudes at a single time point, limiting our ability to infer causality or track changes over time; longitudinal studies are needed. Second, reliance on self-reported data may introduce response and social desirability biases, potentially overestimating positive attitudes. Third, selection bias is possible since those with an interest in digital health may have been more likely to participate, affecting the representativeness of our sample. Additionally, being conducted within the German healthcare system, cultural and technological differences may limit the applicability of our findings to other settings. Finally, we did not collect detailed clinical data on CU subtype or severity. Acceptance of digital health services might differ between chronic spontaneous and inducible urticaria (with varying anaphylaxis risks or symptom control), so future studies should stratify participants by CU subtype and UCT score to guide tailored digital care solutions. While our study provides valuable insights into digital health service perceptions in CU management in Germany, caution is needed when generalizing to other settings, as differences in healthcare infrastructure, cultural attitudes, and digital literacy could significantly influence adoption rates. Further research in diverse contexts is essential to validate and extend these findings [54, 55].

## Conclusion

This study extends the previous study on teledermatology [26] by evaluating digital health literacy, technology readiness, and the acceptance and perceived barriers of digital health services among patients with chronic urticaria (CU) and their healthcare providers. Our findings indicate a generally positive attitude toward digital health tools, with physicians showing greater enthusiasm compared to patients. However, despite the potential of digital platforms to complement conventional care, in-person visits remain crucial—especially for first appointments where handson assessments (e.g., challenge tests) are necessary to confirm diagnosis and guide treatment. Smartphones, the Internet, and email are widely used in CU management, while video consultations and DiGA remain underutilized. Digital tools mainly support communication, symptom monitoring, and prescriptions, but broader integration is needed.

The insights gained here not only refine our digital care model for CU management but also underscore the need for further robust clinical evidence and cost-effectiveness analyses. Future studies should incorporate longitudinal designs, detailed clinical stratification (e.g., CU subtype and UCT scores), and comparisons across diverse healthcare settings to optimize and tailor digital care solutions.

Overall, our study reinforces the potential of digital interventions to enhance chronic urticaria management while highlighting areas for improvement in technology integration and clinical validation.

## Data Availability

All data generated or analyzed during this study are included in this published article. All aggregate data collected for this article are available from the corresponding author upon reasonable request.

## Appendix

**Table:** Sensitivity analysis for'Agree/Strongly Agree (including Neutral)

Question	Frequency (%) Patients
“Do you consider the use of digital health services (e.g., medical apps, video consultations) useful for managing urticaria?”	Agree/Strongly Agree (including Neutral)
Gender	
Divers	1 (0.8)
Male	20 (16.5)
Female	71 (58.7)
Age	
< 30	15 (12.4)
30–49	38 (31.4)
50+	33 (27.3)
Residence	
Small Town	19 (15.7)
Rural Region	20 (16.5)
Medium City	19 (15.7)
Large City	14 (11.6)
Million City	20 (16.5)
Highest Educational Status	
No vocational qualification	1 (0.8)
Currently a student	4 (3.3)
Currently in vocational training	5 (4.1)
Completion of at least one year of vocational training	35 (28.9)
University degree	45 (37.2)
Missing	8 (6.6)
